# Chest Radiographic Patterns and the Transmission of Tuberculosis: Implications for Automated Systems

**DOI:** 10.1371/journal.pone.0154032

**Published:** 2016-04-22

**Authors:** Angela Lau, James Barrie, Christopher Winter, Abdel-Halim Elamy, Gregory Tyrrell, Richard Long

**Affiliations:** 1 Department of Medicine, University of Alberta, Edmonton, Alberta, Canada; 2 Department of Radiology, University of Alberta, Edmonton, Alberta, Canada; 3 School of Public Health, University of Alberta, Edmonton, Alberta, Canada; 4 Department of Laboratory Medicine and Pathology, University of Alberta, Edmonton, Alberta, Canada; 5 Provincial Laboratory for Public Health, Alberta Health Services, Edmonton and Calgary, Alberta, Canada; McGill University, CANADA

## Abstract

**Background:**

Computer-aided detection to identify and diagnose pulmonary tuberculosis is being explored. While both cavitation on chest radiograph and smear-positivity on microscopy are independent risk factors for the infectiousness of pulmonary tuberculosis it is unknown which radiographic pattern, were it detectable, would provide the greatest public health benefit; i.e. reduced transmission. Herein we provide that evidence.

**Objectives:**

1) to determine whether pulmonary tuberculosis in a high income, low incidence country is more likely to present with “typical” adult-type pulmonary tuberculosis radiographic features and 2) to determine whether those with “typical” radiographic features are more likely than those without such features to transmit the organism and/or cause secondary cases.

**Methods:**

Over a three-year period beginning January 1, 2006 consecutive adults with smear-positive pulmonary tuberculosis in the Province of Alberta, Canada, were identified and their pre-treatment radiographs scored by three independent readers as “typical” (having an upper lung zone predominant infiltrate, with or without cavitation but no discernable adenopathy) or “atypical” (all others). Each patient’s pre-treatment bacillary burden was carefully documented and, during a 30-month transmission window, each patient’s transmission events were recorded. Mycobacteriology, radiology and transmission were compared in those with “typical” versus “atypical” radiographs.

**Findings:**

A total of 97 smear-positive pulmonary tuberculosis cases were identified, 69 (71.1%) with and 28 (28.9%) without “typical” chest radiographs. “Typical” cases were more likely to have high bacillary burdens and cavitation (Odds Ratios and 95% Confidence Intervals: 2.75 [1.04–7.31] and 9.10 [2.51–32.94], respectively). Typical cases were also responsible for most transmission events—78% of tuberculin skin test conversions (p<0.002) and 95% of secondary cases in reported close contacts (p<0.01); 94% of secondary cases in “unreported” contacts (p<0.02).

**Conclusion:**

As a group, smear-positive pulmonary tuberculosis patients with typical radiographic features constitute the greatest public health risk. This may have implications for automated detection systems.

## Introduction

Tuberculosis is an illness of great global concern and, in Canada, a high-income, low-incidence setting, the disease persists inequitably among indigenous and foreign-born peoples at rates 34 times and 23 times the rate of Canadian-born non-indigenous persons (Tuberculosis in Canada, 2014 Pre-release). It is an infectious disease spread through aerosolization of the organism via coughing by primarly smear-positive pulmonary tuberculosis (PTB) source cases. As such, the timely identification and treatment of PTB cases is critical to interrupting transmission, optimizing treatment outcome, and meeting elimination targets. In high income, low TB incidence countries such as Canada, a patient may undergo a chest radiograph without any clinical suspicion of PTB. The resultant radiograph requisitions may, therefore, not elicit consideration of PTB by the radiologist. Ideally, the presence of "typical" adult-type PTB radiographic features: a predominantly upper lung zone infiltrate, with or without cavitation, but no discernable intrathoracic adenopathy, should, along with selected pieces of historical information such as community- or country-of-birth, symptomatology, and co-morbidities, prompt consideration of PTB. This is, unfortunately, not always the case, with responsibility for a failure to consider PTB shared between the clinician and the radiologist. If it were possible to automate this process, smear-positive PTB might be diagnosed earlier, transmission reduced and treatment outcomes improved. Herein we investigate the relationship between transmission and “typical” radiographic features to determine the potential public health impact of a more timely diagnosis of PTB via automation of the radiographic assessment.

Our investigations included answering three questions that relate to "typical" adult-type PTB. These are: in a high income, low TB, low HIV incidence jurisdiction, what proportion of smear-positive PTB cases have "typical" versus "atypical" chest radiographic features; what proportion of smear-positive PTB cases with "typical" or "atypical" radiographic features have lung cavitation and/or a high bacillary burden, radiographic and mycobacteriologic features associated with increased transmission; [1–6] and finally what proportion of all transmission events from smear-positive PTB cases are attributable to cases with "typical" versus "atypical" radiographic features. We hypothesize that "typical" cases are more common than "atypical" cases, more likely to have cavitation on chest radiograph and/or a high bacillary burden on mycobacteriology, and more likely to cause transmission events.

## Methods

### Patient characteristics

Over a 36 month period beginning January 1, 2006, sequential adult (age >14 years) smear-positive PTB cases diagnosed in Alberta, a province of Western Canada with a population of 3,645,257 (Statistics Canada, 2011) and a low HIV prevalence, [7] were identified in the Provincial TB Registry.

Each patient’s age, sex and population group (Canadian-born Aboriginal, Canadian-born ‘other’, and foreign-born) was abstracted from public health records. In Canada, Aboriginal includes First Nations (North American Indians), Métis (persons of mixed First Nations and European ancestry) and Inuit (original inhabitants of the far north). ‘High’ and ‘moderate’ risk factors for the development of active TB in persons with presumed latent TB infection (LTBI), as described in the Canadian TB Standards, [8] were identified for each case.

Patient mycobacteriologic histories were abstracted from the Provincial Laboratory for Public Health (PLPH), where all mycobacteriology in the province is performed. Histories included the number, type, semi-quantitative smear size, and time-to-culture-positivity of all smear-positive airway secretion specimens collected within seven days of the start date of treatment. Airway secretion specimens included spontaneously expectorated sputum, induced sputum, auger suction, endotracheal or tracheal tube suctionings, bronchial wash, broncho-alveolar lavage, and airway secretions collected at post-mortem. First-line drug susceptibility testing was performed on all initial isolates of *Mycobacterium tuberculosis*.

### Radiographic features

Posterior-anterior (PA) and lateral (LAT) Digital Imaging and Communications in Medicine (DICOM) chest images, 94% acquired within two weeks of the start date of treatment, were assembled and read by three independent readers (a senior TB pulmonologist and two experienced university-based chest radiologists, one senior and one mid-career). A data abstraction form and accompanying data dictionary was used to report and categorize the chest radiographs (see S1 Fig).

Documented were the presence or absence of: (1) parenchymal infiltrates and their location; for the purpose of this study no distinction was made between infiltrates that were airspace, interstitial, nodular or some combination of these; segments and zones of involvement were recorded; an imaginary horizontal line midway between the apex of the lung and the dome of the diaphragm, divided each lung into two zones, upper and lower; (2) cavities, defined as parenchymal cysts greater than 1 cm in diameter, the widest diameter of the largest cavity and the number of cavities (single or multiple); (3) adenopathy—hilar, mediastinal or both; if parenchymal shadows confluent with the hila or paratracheal mediastinum rendered it impossible to exclude adenopathy, the presence of ‘confluence’ was reported; and (4) pleural effusion.

Information on parenchymal infiltrates, cavitation, adenopathy, and pleural effusion was subsequently used to categorize patients as having “typical” or “atypical” radiographs. For those patients with infiltration localized to or predominantly in the upper lung zones, with or without cavitation, but with no discernable intrathoracic adenopathy, the radiograph was categorized as “typical” for adult-type PTB. [9–11] In patients with: (1) no abnormality; (2) intrathoracic adenopathy with or without parenchymal disease; (3) a localized or predominant lower lung zone infiltrate, with or without cavitation; (4) an isolated pleural effusion; and (5) a miliary (diffuse micronodular) pattern, the radiograph was categorized as “atypical” for adult-type PTB. Extent of disease was coded as minimal, moderately advanced or far advanced according to criteria established by the US National Tuberculosis and Respiratory Disease Association. [12] An inter (between)-reader variability analysis was performed and any discordance resolved by consensus.

### Transmission events

Information on the number, assessment, tuberculin skin test (TST), and disease status of close household and non-household contacts of each PTB case was abstracted from public health records. Complete assessment included a symptom inquiry and tuberculin skin test (TST) 8–12 weeks post-final contact with the source case if not already determined to be TST positive, a chest radiograph if determined to be symptomatic or have a positive TST, and sputum for AFB smear and culture if determined to be symptomatic or have an abnormal chest radiograph. [13] ‘TST conversion’ was defined according to the Canadian TB Standards. [8] Initial isolates of *M*. *tuberculosis* from all culture-positive TB patients in the province between July 1^st^, 2005, i.e. 6 months before the date of diagnosis of the first smear-positive PTB case, and December 31^st^, 2010, i.e. 24 month after the date of diagnosis of the last smear-positive PTB case, regardless of whether or not they belonged to the above smear-positive PTB cohort, were genotyped (see below).

Once PTB cases were identified their contact lists were assembled and cross-referenced against the Provincial Registry to identify any secondary cases. [14] Secondary cases were grouped as *Type 1* or *Type 2* based on their conventional and molecular epidemiologic links to “typical” or “atypical” PTB cases as follows: *Type 1*, individuals diagnosed with active TB within a transmission window that extended from 6 months before to 24 months after the date of diagnosis of the PTB case, listed as a contact of the PTB case, and culture-positive with an isolate of *M*. *tuberculosis* that matched genotypically that of the putative source case; Type 2, individuals notified with active TB within the same transmission window but who were culture-negative (mainly children). The date of diagnosis of the source case was defined as the start date of treatment.

To account for the possibility that PTB cases had incomplete contact lists, secondary cases were searched for among notified cases of TB in the province who were culture-positive, had a genotypically matched isolate of *M*. *tuberculosis*, and were temporally (diagnosed in the same 30-month transmission window) and spatially (lived in the same forward sortation area—a geographic unit associated with a postal facility from which mail delivery originates—as determined by the first three digits of their postal code) linked to the source case. These were termed *Type 3* secondary cases.

Secondary cases that were diagnosed before the date of diagnosis of the source case had to have primary disease. The 30-month transmission window was chosen as the risk of disease after infection is highest during this period of time. [15, 16] Further, it was anticipated that those contacts who were determined to be newly infected but without disease, would be offered treatment of LTBI or alternatively, followed over the subsequent 24 months. “Unreported” contacts, by virtue of being beyond the reach of preventive measures, were theoretically at greater risk of disease (*Type 3*). In the event that a source case was themselves a secondary case of someone else, transmission events attributed to them were scrutinized for plausibility to ascertain whether their “secondary” cases were not more appropriately attributed to their own source case.

### Genotyping methodology

Isolates of *M*. *tuberculosis* from all culture-positive cases of TB diagnosed in the Province of Alberta are routinely fingerprinted using RFLP, supplemented in those isolates with five or fewer copies of the insertion sequence *6110*, by spoligotyping. [17, 18] The analysis is performed on coded specimens in a blinded fashion. The images are digitized using an imager video camera system, and subsequently analyzed in a blinded fashion using the Gelcompar II software. To improve accuracy, all isolates matched as identical by the computer were manually confirmed by visual comparison of the original autoradiographs. Over the six months preceding the study period, the three year study period, and the two years following the study period (5.5 years), a total of 784 cases of TB were diagnosed in the Province of Alberta of which 652 (83.2%) grew *M*. *tuberculosis* and 650 (99.7%) were genotyped.

### Statistical analysis

IBM SPSS Statistics software version 17.0 was used for analysis of the data. Generalized kappa statistics were performed to quantify the level of agreement between radiograph readers, with the standard error reported as the asymptotic variance. Associations between the demographic and mycobacteriologic characteristics of “typical” versus “atypical” cases were evaluated with either binary or multi-nominal logistic regression. The logistic regression was used to estimate the odds ratio (OR) of characteristics of “typical” versus “atypical” cases, along with their 95% confidence interval (95% CI). Univariate analysis was performed for categorical data, including the analysis of transmission events, using the Pearson’s chi-squared test or the Fisher’s exact test as appropriate. A two-tailed p-value <0.05 was taken as statistically significant. For comparison of the mean number of assessed contacts per case and the mean number of days to assessment of TST converters, a t-test was employed.

Study approval was obtained from the University of Alberta Health Research Ethics Board (HREB). Retrospective analysis of anonymous and routinely collected surveillance data did not require direct patient contact; therefore the need for patient’s informed consent was waived by HREB.

## Results

Between January 1, 2006 and December 31, 2008, 99 adult (age >14 years) smear-positive PTB patients were diagnosed in the province of Alberta, Canada, and notified in the Provincial TB Registry. Two patients were excluded from the analysis; one because a computed tomographic scan alone was available; another because the plain chest radiograph was technically inadequate for interpretation.

**Fig 1:**
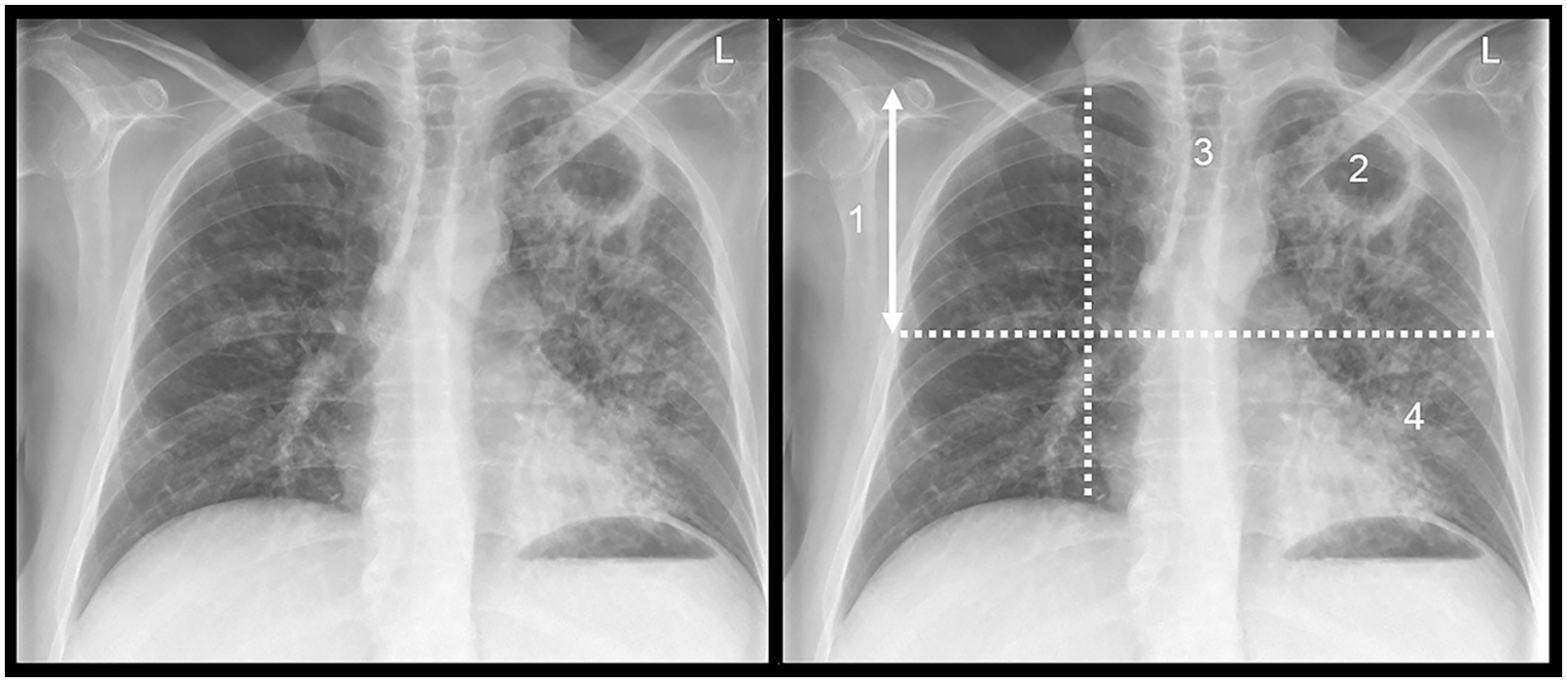
A posterior-anterior chest radiograph in a patient with typical adult-type smear-positive pulmonary tuberculosis. The major features are: (1) upper lung zone distribution; (2) cavitation; (3) volume loss; (4) acinar shadows.

The analysis of inter-reader variability included disease type (“typical” vs “atypical”) as well as those radiographic features judged to most accurately reflect the pathology of post-primary PTB in an immunocompetent host: (i) distribution of disease (predominantly upper lung zone), (ii) cavitation, (iii) volume loss, and (iv) poorly defined nodules (acinar shadows); see Fig 1. [19–21] Kappa statistics showed substantial agreement (>0.60) for disease type, distribution, and cavitation, and fair agreement (<0.3) for volume loss and poorly defined nodules (see Table 1). [22]

**Table 1:** Expert inter-reader variability of chest radiographic interpretations.

Expert reader interpretation*	Agreement	Kappa statistic	Asymptotic standard error (ASE)
**Disease type (“typical” vs “atypical”)**	Substantial	0.660	0.082
**Presence or absence of cavitation**	Substantial	0.749	0.067
**Presence or absence of upper lung zone disease**	Substantial	0.643	0.097
**Presence or absence of volume loss**	Fair	0.352	0.074
**Presence or absence of acinar shadows**	Fair	0.257	0.059

*See text for definition of terms.

Of the 97 cases that were included in the analysis, 69 (71.1%) had “typical” and 28 (28.9%) had “atypical” chest radiographs. Patients with “typical” and “atypical” chest radiographs did not differ by age, sex, or population group (Table 2). HIV co-infected patients were more likely to have “atypical” chest radiographs (p = 0.007); cases with other risk factors were no more likely to have “typical” than “atypical” radiographs.

**Table 2:** Demographic and clinical features of smear-positive PTB patients with “typical” and “atypical” chest radiographic features.

Patient Demographics and Clinical Features	Total No. (%)	CXR Category		OR (95% CI)
		“Typical” No. (%)	“Atypical” No. (%)	
**No. Assessed**	97 (100.0)	69 (71.1)	28 (28.9)	
**Age**				
15–64	71 (73.2)	54 (78.3)	17 (60.7)	1.0
≥65	26 (26.8)	15 (21.7)	11 (39.3)	0.43 (0.17–1.11)
**Sex**				
Male	53 (54.6)	40 (58.0)	13 (46.4)	1.0
Female	44 (45.4)	29 (42.0)	15 (53.6)	0.63 (0.26–1.52)
**Population Group**				
CBA and CBO	30 (30.9)	22 (31.9)	8 (28.6)	1.0
FB	67 (69.1)	47 (68.1)	20 (71.4)	0.86 (0.33–2.24)
**HIV Status**				
Negative	73 (75.3)	56 (81.2)	17 (60.7)	1.0
Unknown	10 (10.3)	8 (11.6)	2 (7.1)	
Positive	14 (14.4)	5 (7.2)	9 (32.1)	0.17 (0.05–0.55)
**Other Risk Factors**				
None or Unknown	44 (45.3)	29 (42.0)	15 (53.6)	1.0
1 or more	53 (54.6)	40 (58.0)	13 (46.4)	1.59 (0.66–3.85)

Abbreviations: PTB pulmonary TB; CXR chest radiograph; CI confidence interval; CBA Canadian-born Aboriginal; CBO Canadian-born ‘other’; FB foreign-born.

The bacillary burden and radiographic features of PTB cases at the time of diagnosis are described in Table 3. The number of specimens collected per case was not statistically significantly different among the “typical” and “atypical” cases. All positive smears were 1+ or greater; all 3+ or greater smears had >10 acid-fast bacilli (AFB) per high power field using Fuchsin stain [8]. Compared to “atypical” cases “typical” cases were more likely to have semi-quantitative smear sizes of 3+ or greater. Consistent with this higher bacillary burden “typical” cases were more likely to have pre-treatment specimens with shorter times-to-liquid culture positivity, ≤ 1 week vs >1 week (p = 0.03); time-to-liquid culture positivity being understood to be a quantitative measurement of metabolic activity inversely related to the number of viable bacilli inoculated [23, 24]. Initial isolates from “typical” cases were more likely than initial isolates from “atypical” cases to be drug-resistant (13.0% vs 3.6%) though the difference was not statistically significant. Patients with “typical” chest radiographs were more likely than those with “atypical” chest radiographs to have moderately advanced or far advanced disease and to have lung cavitation. Semi-quantitative smear size did not differ in “typical” versus “atypical” cases after adjustment for cavitation (p = 0.580).

**Table 3:** Bacillary burden and cavitation in smear-positive pulmonary TB cases with “typical” and “atypical” chest radiographic features.

Mycobacteriologic and Radiographic Features	CXR Category		OR (95% CI)
	“Typical” No. (%) (n = 69)	“Atypical” No. (%) (n = 28)	
**Number of Specimens Collected**			
1 or 2	32 (46.4)	19 (67.9)	1
≥3	37 (53.6)	9 (32.1)	2.44 (0.97–6.15)
**Semi-quantitative Smear Size**			
<3+	36 (52.2)	21 (75.0)	1
≥3+	33 (47.8)	7 (25.0)	2.75 (1.04–7.31)
**Time-to-culture Positivity (Days)**			
Less than one week	39 (56.5)	9 (32.1)	1
One week or greater	30 (42.5)	19 (67.9)	0.36 (0.14–0.92)
**Drug Resistance**			
No Drug Resistance	60 (87.0)	27 (96.4)	1
Drug Resistance*	9 (13.0)	1 (3.6)	4.05 (0.49–33.58)
**Extent of Disease**^†^			
Minimal or other	14 (20.3)	20 (71.4)	1
Moderately advanced	30 (43.5)	7 (25.0)	9.82 (3.58–26.92)
Far advanced	25 (36.2)	1 (3.6)	
**Cavitation**			
No Cavitation	33 (47.8)	25 (89.3)	1
Cavitation Present	36 (52.1)	3 (10.7)	9.10 (2.51–32.94)

Abbreviations: PTB pulmonary TB; CXR chest radiograph

*8 “typical” cases and 1 “atypical” case had isoniazid resistance

^†^See text and reference #12.

The number of close contacts identified and assessed per PTB case was similar in “typical” and “atypical” cases (p = 0.789 and p = 0.257, see Table 4). “Typical” cases had more TST converters than “atypical” cases (p = 0.002); time to assessment of both groups was similar (p = 0.462). “Typical” cases also had more secondary cases than “atypical” cases (24 vs 1, p = 0.01); and if each secondary cases is also considered to be a converter then TST converters of “typical” cases were much more likely than TST converters of “atypical” cases to be secondary cases (p = 0.001). Most secondary cases were diagnosed within six months of the source case and therefore were considered co-prevalent (20 of 24 [83.3%] secondary cases of “typical” cases and 1 of 1 [100%] secondary cases of “atypical” cases). [16] Among “typical” cases, TST converters of cavitary cases were twice as likely as TST converters of non-cavitary cases to be secondary cases (47.2% vs 22.6%), see Table 5.

**Table 4:** Transmission events among close contacts of smear-positive PTB Cases according to chest radiograph category.

		CXR Category		
Characteristic	Total	Typical (n = 69)	Atypical (n = 28)	p-value
No. Contacts Identified	1442	1000	442	0.789
No. Contacts Assessed (% of those identified)	1161 (80.5)	813 (81.3)	348 (78.7)	0.257
No. Assessed Contacts Per Case (Mean±SD)	12.0±17.6	12±18.2	12±16.4	0.861
No. Contacts with TST Conversion*	86	67	19	0.002
No. Days to Assessment of TST Converters (Mean±SD)	90.2±102.3	85.8±92.1	105.5±134.2	0.462
No. of TST Converters Who Were Secondary Cases^†^	25	24	1	0.010
No. of Secondary Cases Per TST Converter (Attack Rate)	0.29	0.36	0.05	0.001

Abbreviations: PTB pulmonary TB; CXR chest radiograph; No. number; TST tuberculin skin test

* No. of contacts with TST conversion include *Type 1* and *2* secondary cases

^**†**^ Two secondary cases were listed as casual/medium risk contacts of “typical” cases.

**Table 5:** Transmission Events in Close Contacts of “Typical” and “Atypical” Pulmonary TB Cases grouped according to Radiographic Appearance (Cavitary or Non-Cavitary) and Sputum Semi-quantitative Smear Size.

Number of Cases	CXR (Cavitation)	Smear Size*	Transmission Events		“Attack Rate” ^†^ (%)
			Converters Alone (n)	Converters Secondary Cases (n)*	
**Typical**					
25	Yes	≥ 3+	16	14	46.7
11	Yes	< 3+	3	3	50.0
8	No	≥ 3+	4	1	20.0
25	No	< 3+	20	6	23.1
**Atypical**					
1	Yes	≥ 3+	1	0	0
2	Yes	< 3+	2	0	0
6	No	≥ 3+	3	0	0
19	No	< 3+	12	1	7.7

Abbreviations: CXR chest radiograph

* see text for definition of smear size and *Type 1* and *Type 2* secondary cases

^†^ “Attack Rate” = secondary cases/converters that were or were not secondary cases.

Conventional and molecular epidemiology identified a total of 42 secondary cases, 17, 8, and 17 *Type 1*, *2*, *and 3*, respectively (see Table 6). Compared to “atypical” cases, “typical” cases were responsible for more secondary cases of all three types (p = 0.001). When broken into subcategories, “typical” cases were found to have significantly more *Type 1* and *Type 3* secondary cases (p = 0.002 and 0.020, respectively). When all transmission events were considered it was noted that less than 50% of source cases, regardless of chest radiograph category, had one or more transmission event (47.8% and 46.4%, respectively) (see Table 7). “Typical” cases included 92.3% of those with lung cavitation. Among “typical” cases the presence of cavitation and a larger smear size were associated with transmission; among “atypical” cases the presence of cavitation was associated with transmission, though these differences were not statistically significant, probably because the numbers are small.

**Table 6:** Secondary cases among smear-positive PTB patients according to chest radiograph category.

Secondary Cases by Type*	Total	CXR Category		*p*-value*
		Typical (n = 69)	Atypical (n = 28)	
*Type 1*	17	17	0	0.002
*Type 2*	8	7	1	0.420
*Type3*	17	16	1	0.020
*All Types*	42	40	2	0.001

Abbreviations: PTB pulmonary TB; CXR chest radiograph; TST tuberculin skin test interval

*Median (Range) days until diagnosis of *Type 1*, *2*, *and 3* cases were 52 days (4–507 days), 28 days (4–91 days), and 433 days (50–730 days), respectively.

**Table 7:** Transmission events by source case characteristic and chest radiograph category.

Source Case Characteristic*	Radiograph Category					
	Typical		OR (95% CI)	Atypical		OR (95% CI)
	with transmission events (n = 33)	without transmission events (n = 36)		with transmission events (n = 13)	without transmission events (n = 15)	
**Age (years)**						
15–64	27 (81.8)	27 (75.0)	1.00	8 (61.5)	9 (60.0)	1.00
≥65	6 (18.2)	9 (25.0)	0.67 (0.21–2.13)	5 (38.5)	6 (40.0)	0.94 (0.20–4.29)
**Smear Size**						
≥3+	19 (57.6)	14 (38.9)	1.00	2 (15.4)	5 (33.3)	1.00
<3+	14 (42.4)	22 (61.1)	0.47 (0.18–1.23)	11 (84.6)	10 (66.7)	2.75 (0.43–17.49)
**Drug Resistance**						
No	30	33	1.0	13	14	1.00
Yes	3	6	0.55 (0.13–2.40)	0	1	0.36 (0.01–9.57)
**Cavitation**						
No	12 (36.4)	21 (58.3)	1.00	10 (76.9	15 (100.0)	1.00
Yes	21 (63.6)	15 (41.7)	2.45 (0.93–6.47)	3 (23.1)	0 (0.0)	10.33 (0.48–221.50)

*Smear size refers to the largest semi-quantitative smear size at microscopy; drug resistance refers to resistance to one or more first-line anti-tuberculosis drugs.

## Discussion

Making a timely diagnosis of pulmonary TB in high income countries where the disease is not common or in middle to low income countries where technological advances or skilled workers may be in short supply is a daunting task. If, through inexperience or inadequate human resources, a diagnosis of PTB is delayed or not made by the clinician or the radiologist, transmission is ongoing and outcomes potentially poorer. In our study there was substantial inter-observer agreement on what constituted “typical” versus “atypical” chest radiographic features of adult-type PTB. After radiograph characterization, we found that “typical” cases were more common than “atypical” cases, accounting for over two-thirds of all PTB cases in Alberta, an immigrant-receiving province of Western Canada. [25]

“Typical” cases were also more likely to have cavitations on chest radiograph and/or high bacillary burdens. Cavitary disease and bacillary burden are known to be highly correlated; caseous necrosis allowing for extracellular replication, amplifying the bacterial load. Each is considered to be an independent risk factor for transmission. [1–6] Given these findings, it is not surprising that we found “typical” cases had more transmission events than “atypical” cases, accounting for 78% of TST conversions and 95% of secondary cases. “Typical” cases were also more likely than “atypical” case to be harbouring a drug-resistant strain though the difference was not statistically significant. Some data suggests that, compared to drug-susceptible strains, drug-resistant strains are less transmissible. [26, 27] Our results suggest that a computer-aided detection system focused on radiographic features of “typical” adult-type PTB and linked to clinical data may have public health utility. [28–31]

In his widely acclaimed book “*Tuberculosis*: *Cases Finding and Chemotherapy*: *Questions and Answers*”, Toman concluded that smear-negative culture-positive PTB and smear-positive culture-positive PTB were different phenotypic expressions of the same disease; [32] smear-negative disease being symptomatic 50% of the time and resulting in intermittent excretion of small numbers of bacilli; smear-positive disease developing within the same timeframe as smear-negative disease but being symptomatic 90% of the time and much more infectious—subsequently proven to be at least five times more infectious. [33, 34] Without treatment minimal smear-negative disease did not necessarily progress to advanced smear-positive disease.

Less well known is the fact that TST converters of smear-positive cases are much more likely than TST converters of smear-negative cases to be secondary cases, [35] an outcome that is best explained by the greater likelihood of reinfection occurring from smear-positive cases and the delayed maturation of cell mediated immunity after the initial infection. That reinfection within the first 18 months of an initial infection is much more likely to cause disease than reinfection that occurs later, was strongly suggested by R.G. Ferguson in the pre-antibiotic era. [36] Many subsequent “experiments-of-nature” such as the well documented outbreak of TB on the U.S. naval vessel *Richard E*. *Byrd*, strongly support Ferguson’s findings. [37, 38] In our own study we found that 24 out of 67 TST converters of “typical” cases (36%), versus 1 of 19 TST converters of “atypical” cases (5%), were secondary cases. We also found that most of the secondary cases were co-prevalent and therefore beyond the reach of preventive measures. Conceivably cavitation and a high bacillary burden in smear-positive “typical” cases results in a higher probability of reinfection and disease than in smear-positive “atypical” cases.

If indeed a gradient of infectiousness exists within smear-positive PTB cases, “typical” smear-positive cases being more infectious than “atypical” smear-positive cases, and if indeed the detection of the radiographic features of “typical” cases can be readily automated, then there is the possibility that “typical” cases can be diagnosed earlier and the public health consequences of their disease reduced. Cough aerosol studies would be required to confirm the “typical” vs “atypical” gradient and shed further light on the heterogeneity of the “typical” group with respect to their ability to transmit—only 47% of the “typical” cases had a transmission event, [39] though such heterogeneity is not inconsistent with other studies. [40, 41]

Strengths of our study include the inclusion of consecutive smear-positive PTB cases, the categorization of case-patient radiographs by three independent readers using a standardized data abstraction tool, the systematic assessment of case contacts according to provincial protocol, and the thorough application of conventional and molecular epidemiologic tools to identify secondary cases from among reported and “unreported” contacts. Limitations of our study include the retrospective study design (sputum samples, for example, were not systematically collected, nor was information on cough, though cough frequency is known to be less powerful than sputum microscopy or chest radiograph in predicting infectivity), [42] and the relatively small sample size.

## Conclusions

After identifying consecutive adult cases of smear-positive PTB in our jurisdiction, we created a chest radiograph library and sorted the cases according to whether or not they had radiographic features “typical” of adult-type PTB. Inter-observer agreement on the “typical” versus “atypical attribution of radiographs was substantial. We then determined that those with “typical” chest radiographic features were not only more common, but more likely to be cavitary/high bacillary burden and to transmit than those with “atypical” chest radiographic features. Cumulatively, “typical” cases accounted for 78% of the TST conversions and 95% of the secondary cases. These results suggest that computer-aided detection systems that focus on “typical” adult-type PTB may have the greatest public health benefit.

## Supporting Information

S1 Fig Data abstraction form. Data abstraction form and dictionary used to categorize patients as being typical (post-primary) or atypical (primary or indeterminate).
(DOCX)
