# Supplementary material for: Chest Radiographic Patterns and the Transmission of Tuberculosis: Implications for Automated Systems
Source: PLoS One. 2016 Apr 22;11(4):e0154032. doi: 10.1371/journal.pone.0154032 (PMC4841548; doi:10.1371/journal.pone.0154032)

**S1 Fig.** **Data abstraction form.** Data abstraction form and dictionary used to categorize patients as being typical (post-primary) or atypical (primary or indeterminate).


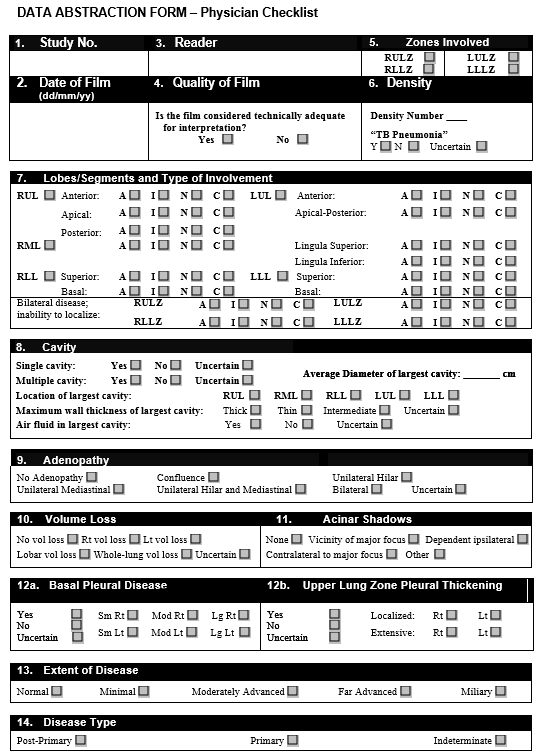


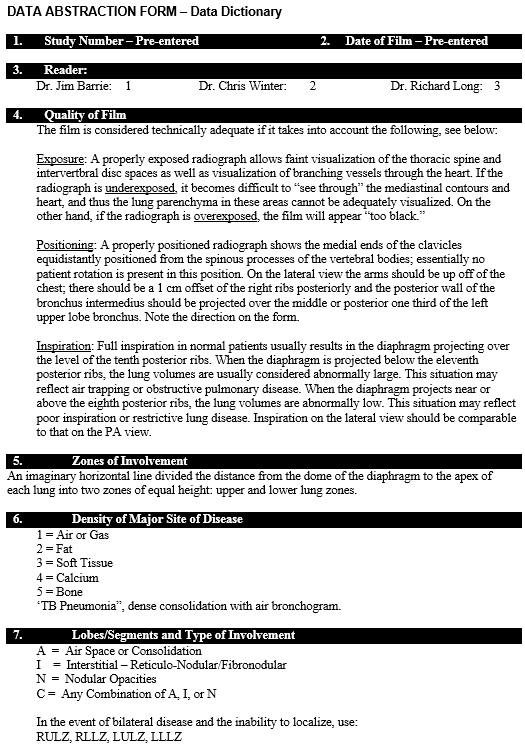


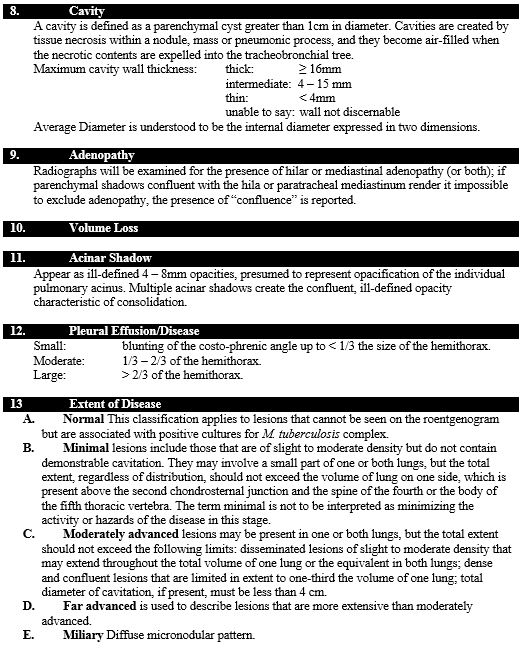


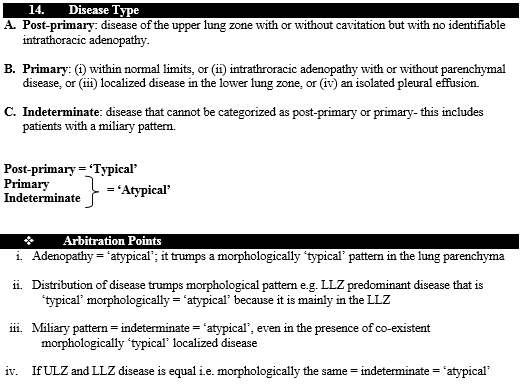

Supplement: S1 Fig — Data abstraction form and dictionary used to categorize patients as being typical (post-primary) or atypical (primary or indeterminate). (DOCX) [file pone.0154032.s001.docx]
